# Biomechanical comparison of different framework materials in mandibular overdenture prosthesis supported with implants of different sizes: a finite element analysis

**DOI:** 10.1186/s12903-023-03080-1

**Published:** 2023-07-05

**Authors:** Elifnur Güzelce S

**Affiliations:** Department of Prosthodontics, University of Health Sciences Turkey, Hamidiye Campus (Istanbul) Selimiye Mah. Tıbbiye Cad. No:38, Üsküdar/Istanbul, 34668 Türkiye

**Keywords:** Finite element analysis, Implant supported overdenture, Mini implants, PEEK

## Abstract

**Background:**

The aim of this study is to evaluate the stresses on the supporting bone, implants, and framework materials under masticatory forces in mandibular overdenture prostheses modeled with different framework materials and different implant types, using the Finite Element Analysis (FEA).

**Methods:**

For the finite element modeling, two identical mandibular jaw models were created; one with two standard (diameter:4.1 mm/12 mm length) and the other with two mini-implants (diameter:2.4 mm/12 mm length) were placed in the canine teeth area. The polymethylmethacrylate (PMMA) denture was modeled upon them, supported by Cobalt Chromium alloy (CoCr), Poly-ether ether ketone (PEEK), and Zantex materials with framework. No framework was added as a control model; only PMMA overdenture prosthesis was modeled.

**Results:**

Regardless of the framework materials of the overdenture prostheses, the stress values ​​on mini-implants in all models yielded approximately two times higher results comparing to standard implants. More stress transmission was observed in the supporting bone and implants in the control prostheses and overdenture prostheses supported with respectively PEEK, Zantex, CoCr alloy frameworks, respectively. In the framework materials, more stress occurred on CoCr, Zantex and PEEK in that order.

**Conclusion:**

In the light of this study, the use of mini-implants as an alternative to standard implants is not promising in terms of distribution and transmission of chewing stresses. As a framework material, standard rigid metal alloys were found to be more advantageous than polymer materials in terms of stress distribution.

## Background

One of the biggest problems in edentulous patients is the stability, retention, and mobility of removable complete dentures. In addition to these problems, pain, speech difficulties, and chewing problems are also seen in patients who use conventional mandibular dentures with excessive bone loss [1]. Implant-supported overdenture prostheses significantly increase the quality of life of patients who are not satisfied with removable complete denture. Overdenture prosthesis treatment supported by two implants in the edentulous mandible is recommended as a standard treatment protocol [2, 3]. In recent studies, mini dental implants have also been used to support overdenture prostheses in mandibular arch with severe bone resorption and in patients who cannot handle surgical procedures [1–4]. Mini implants are generally produced in diameters less than 3 mm and as a one-piece system. Comparing to traditional implants, they are less invasive, surgically placed on the jawbone, has fewer complications, and they are cheaper and more comfortable [2, 4, 5]. There are studies using 2 or 4 implants to support overdenture prostheses [1, 2, 5].

Implant supported overdenture prostheses tend to deform when used in weak points such as the abutment perimeter and midline. It has been stated that supporting the overdenture denture base with a cast metal framework would be effective in dispersing chewing stresses and preventing base fractures [6]. However, metal alloys have disadvantages such as difficulty in construction, risk of allergies, metallic color and metallic taste. In this regard, there are studies where the denture base is supported by fiber materials. Fiber materials are a simpler and more aesthetic option than metal framework materials [7–9]. Metal frameworks and Poly-ether ether ketone (PEEK) materials are used as prosthetic base materials. Furthermore, recent polymer materials that are lighter than metal produced with CAD/CAM and that have a modulus of elasticity closer to bone have been used as an alternative to metal frameworks [6, 10, 11].

PEEK is a semi-crystalline organic polymer with high chemical and mechanical properties [10, 12]. As an alternative to patients with metal allergies, PEEK is used in prosthetic treatments due to its high heat resistance, biocompatibility, resistance to oral fluids and ease of use. It has mechanical properties similar to dentin [6, 12]. PEEK material can be combined with ceramic materials and fiber materials to improve its strength or aesthetic properties [13, 14]. Removable denture bases can be produced from PEEK using injection molding or CAD/CAM systems [14]. PEEK material can be an alternative to metal frameworks in removable prostheses [12]. Zantex is a material containing a high-performance polymer matrix with three-dimensional dense glass fiber added. It has mechanical properties close to bone and is biocompatible.The modulus of elasticity is lower than CoCr alloy and higher than PEEK material. With composite and PMMA, connections can be established by sandblasting and bonding and by milling and adding directly. It is recommended that the areas in contact with the gums be covered with a glaze [15]. It demonstrates high fracture resistance when used as a framework material in partial and removable complete dentures. As Zantex is a newly produced material, the studies in this respect is still quite limited [11, 15, 16].

Finite element analysis (FEA), is a reliable and convenient method to evaluate stress occurring around the bone and implant. This analysis can be used not only to obtain baseline data for new methods used in the clinic, but also to determine potential effects. This analysis generates computational data that reveal the behavior of new materials or techniques under simulated clinical conditions [17].

To the authors’ knowledge, no study has been published in which polymer framework materials (PEEK, Zantex) that have recently appeared on the market in overdenture prostheses made on mini and standard implants are compared with metal frameworks Cobalt Chromium alloy (CoCr) and acrylic base without framework Polymethylmethacrylate (PMMA). Additionally, there are no publications on framework material for removable prostheses in relation to Zantex. The null hypothesis was that standart implant supported overdenture prostheses reinforced by polymer materials create lesser stress on the bone than metal frameworks.

## Methods

In this study, FEA models with two dental mini-implants (M1) (Straumann, Institute Straumann AG, Basel, Switzerland) and two standard implants (M2) placed in the canine tooth area were created on the same two mandibular bone models. Four overdenture prostheses with PMMA, PEEK, Zantex (Biofunctional materials, Florida, USA), and CoCr frameworks without a framework were modeled with FEA.

(M1-PMMA, M1-PEEK, M1-Zantex, M1-CoCr; M2-PMMA, M2-PEEK, M2-Zantex, M2-CoCr).

**Fig. 1:**
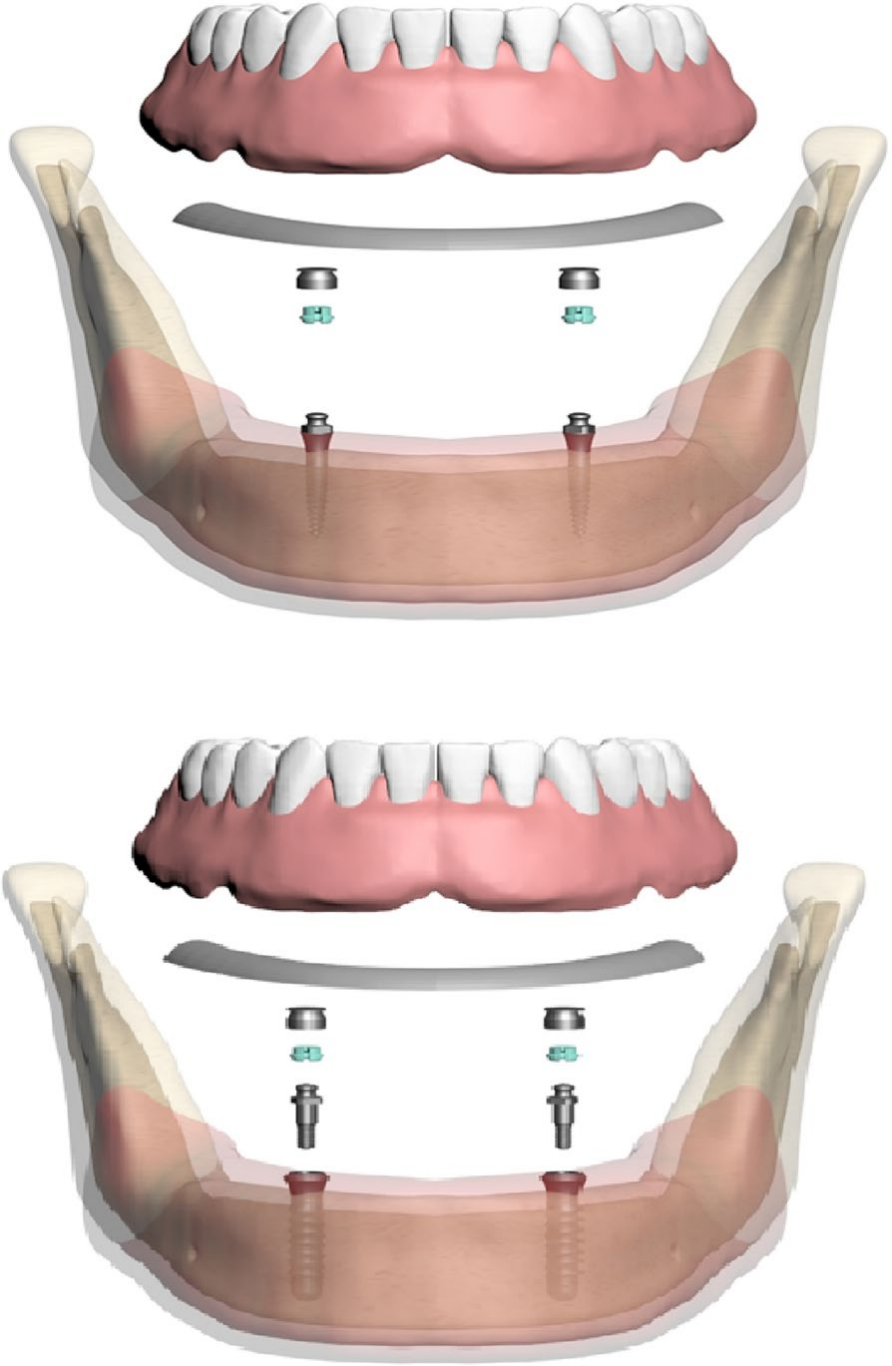
Overview components of the FEA models

M1-(2.4 mm diameter/12 mm length) conventional implant M2- (4.1 mm diameter/12 mm length). Straumann one-piece mini-implant implants were planned. The O-ring ball abutment was modeled on conventional implants. In the overdenture models, the framework materials were modeled as 1 mm PMMA at the top -0.5 mm framework material in the middle, with -0.5 mm PMMA at the bottom. The framework materials were modeled along the mandibular crest. Von misses and principle stresses on implants, framework materials, and supporting bone were evaluated with FEA. The created models and their components are shown in Fig. 1.

### Modeling of bones

The mandibular bone model used in the study is based on the tomography scan of a completely edentulous adult individual. Tomography data were reconstructed with a slice thickness of 0.1 mm. The tomography data obtained as a result of the reconstruction were transferred to 3DSlicer software (2023, Slicer Community. Revision a823405b). in DICOM (DCM) format. CT data in DICOM format were separated according to appropriate Hounsfield values ​​in 3DSlicer software and converted into a three-dimensional model by segmentation. The model was exported in STL format. The three-dimensional CAD model was imported into ALTAIR Evolve (ALTAIR, Troy, MI, USA) software, where the appropriate mandibular bone was designed. Trabecular bone was obtained by taking the inner surface of the three-dimensional cortical bone of the mandible as a reference, the thickness of which was adjusted. A 2 mm thick mucosa was modeled with reference to the outer surface of the cortical bone. Bone tissues were considered to be isotropic, linear, homogeneous, and %100 osseointegrated into the implants [18, 19]. The denture and implant were provided with bonded contact for all models. A flat surface was created underneath and boundary conditions were applied to stabilize the base of the mandible. 

### Modeling of implants and overdentures

The implants used in the study were modeled using ALTAIR Evolve software based on the measurements. The three-dimensional scanning process of the created overdenture model was performed with a Panda P2 (Pingtum, Suzhou, CHINA) scanning device. To ensure force transfer between the models, the matching process was performed between the mesh structures. The material properties of the analyzed model are defined numerically [1, 6, 15] (Table 1).

**Table 1:** Elastic modulus and Poisson’s ratios of the materials used in the study

Materials	Young modulus (GPa)	Poisson's ratio	References
Mini and conventional implants/ball atachment/housing (Ti 6Al 4 V)	110	0,35	[6]
Trabecular bone	1,37	0,30	[6]
Cortical bone	13,7	0,30	[6]
Nylon	0,005	0,4	[1]
Mucosa	0,34	0,45	[6]
Acrylic resin teeth	2,94	0,30	[6]
Acrylic resin base	1,96	0,30	[6]
Cobalt- chromium cast metal	275	0,33	[15]
PEEK	4	0,40	[15]
ZANTEX	35	0,40	[15]

### Creation of mathematical models

Mathematical models were formed by dividing geometric models into simple and small pieces called meshes. After the modeling process was completed in the ALTAIR Evolve software, the models were mathematically created with ALTAIR Hypermesh software and made ready for analysis. Models prepared in ALTAIR Hypermesh software were transferred to the ALTAIR OptiStruct analysis program in.FEM format to perform the analysis. Quantitative model information for the two different analysis models created is depicted in Table 2.

**Table 2:** Quantitative model information of FE

Nodes and Elements	M1	M2
Total of Nodes	495,906	651,659
Total of Elements	1,982,471	2,634,695

### Loading Scenarios and Boundary Conditions

**Fig. 2:**
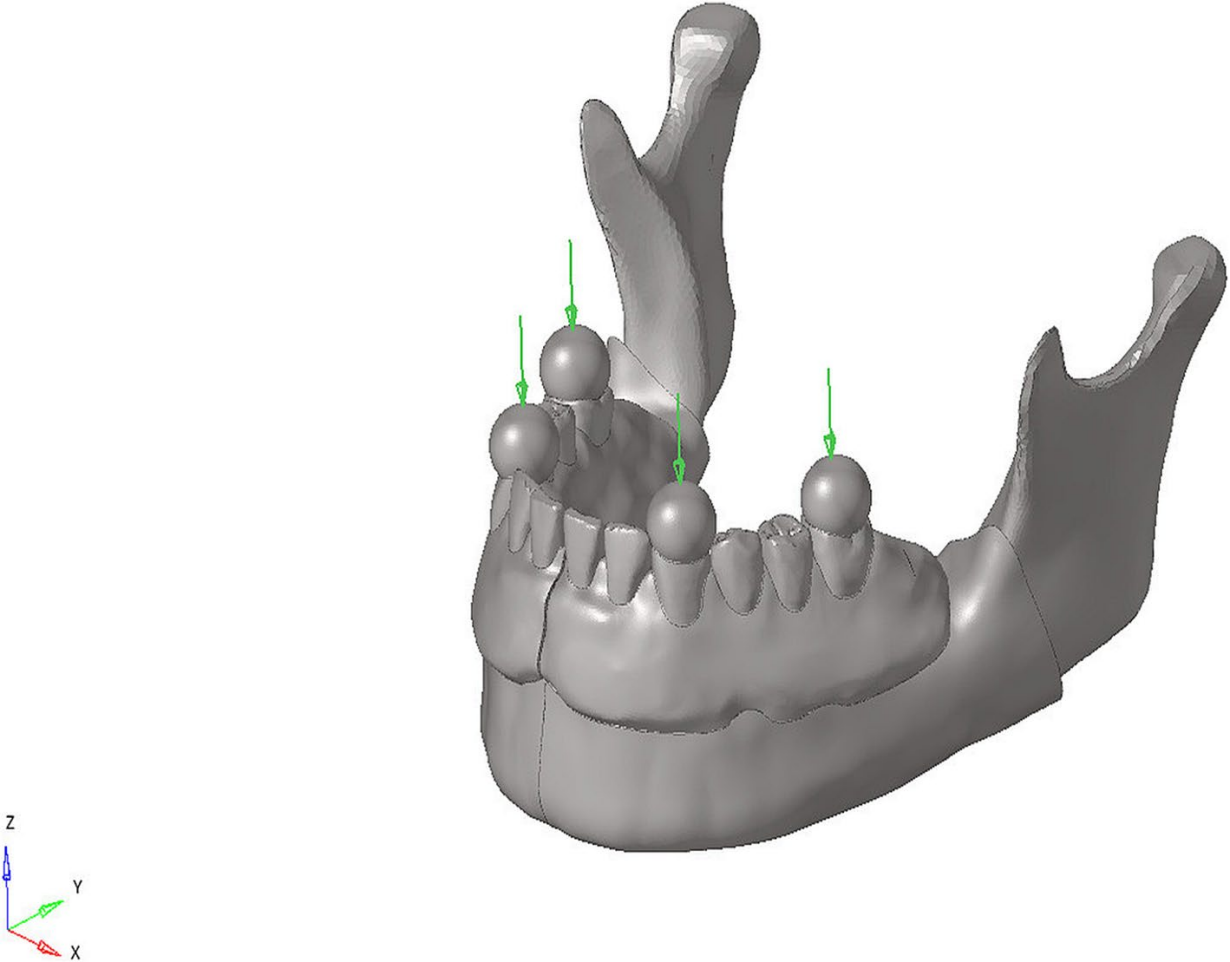
Example of loading conditions

For both models, a load of 50 N was applied at a 90-degree angle over the foodstuff to simulate the chewing force on the canine and first molars. In total of six linear static analyses were carried out for the two models under the single loading condition. By using foodstuff in the chewing force simulation, the loads were distributed and the stress singularity was prevented in the loading zones. The foodstuff force application scenario is shown in Fig. 2.

The models were fixed by restricting all degrees of freedom from the nodal points in the lower regions of the cortical bone and mucosa, preventing movement in all three axes. Boundary condition was applied to all parts in the model so that the X axis is symmetrical with respect to the Y–Z plane.

## Results

**Fig. 3:**
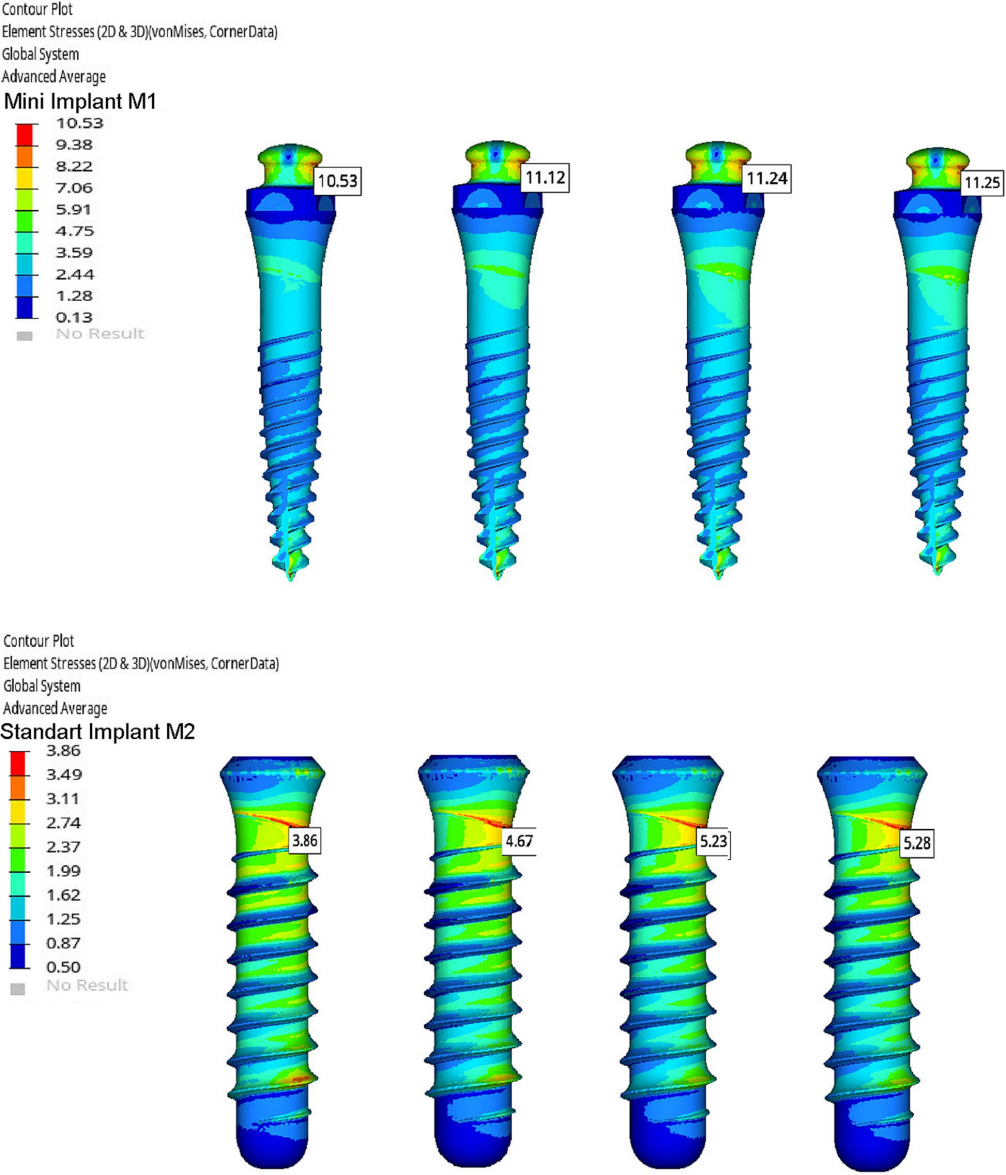
Von Mises Stresses (N/mm2) of implants

**Fig. 4:**
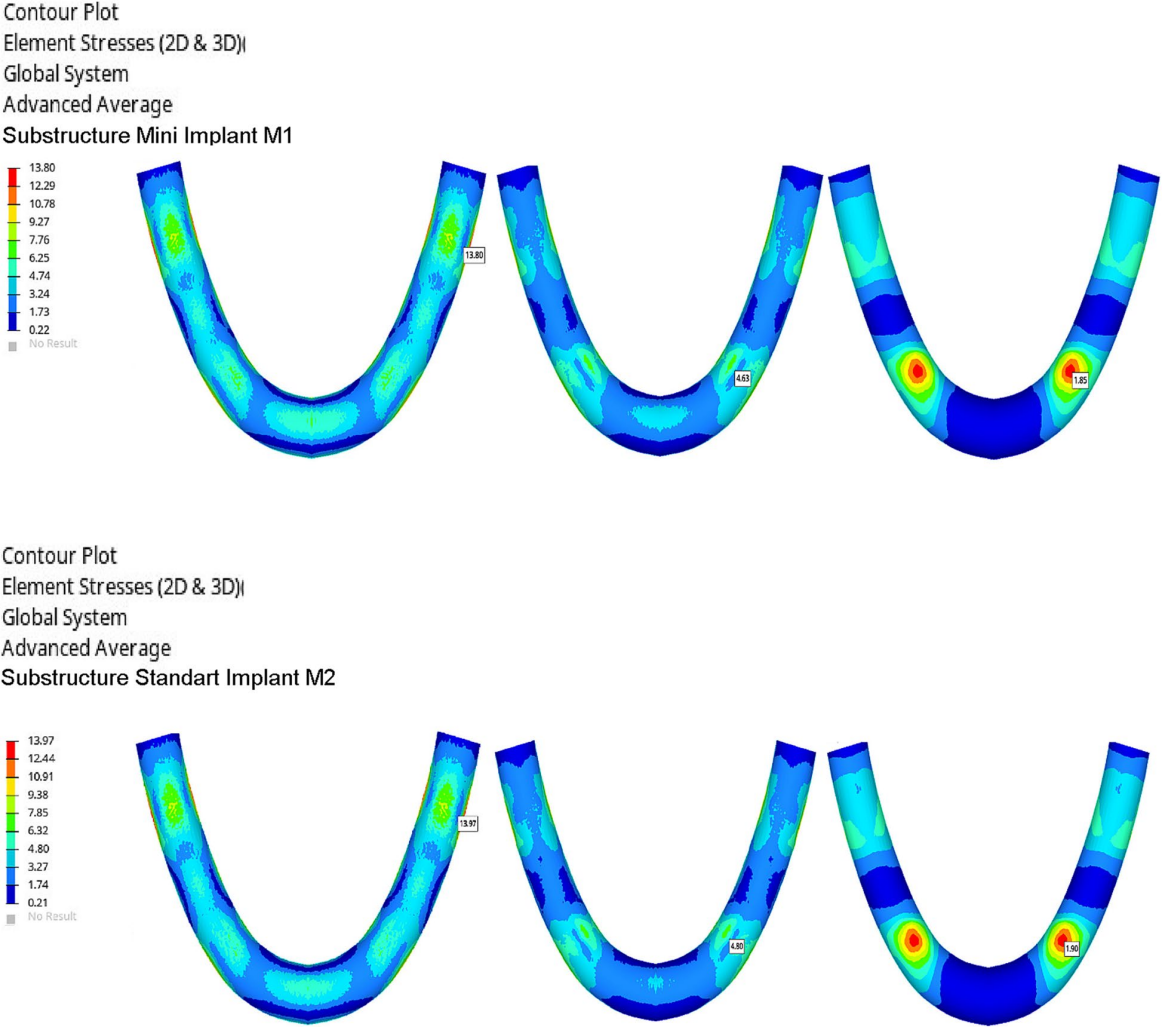
Von Mises Stresses (N/mm2) of substructure materials

**Fig. 5:**
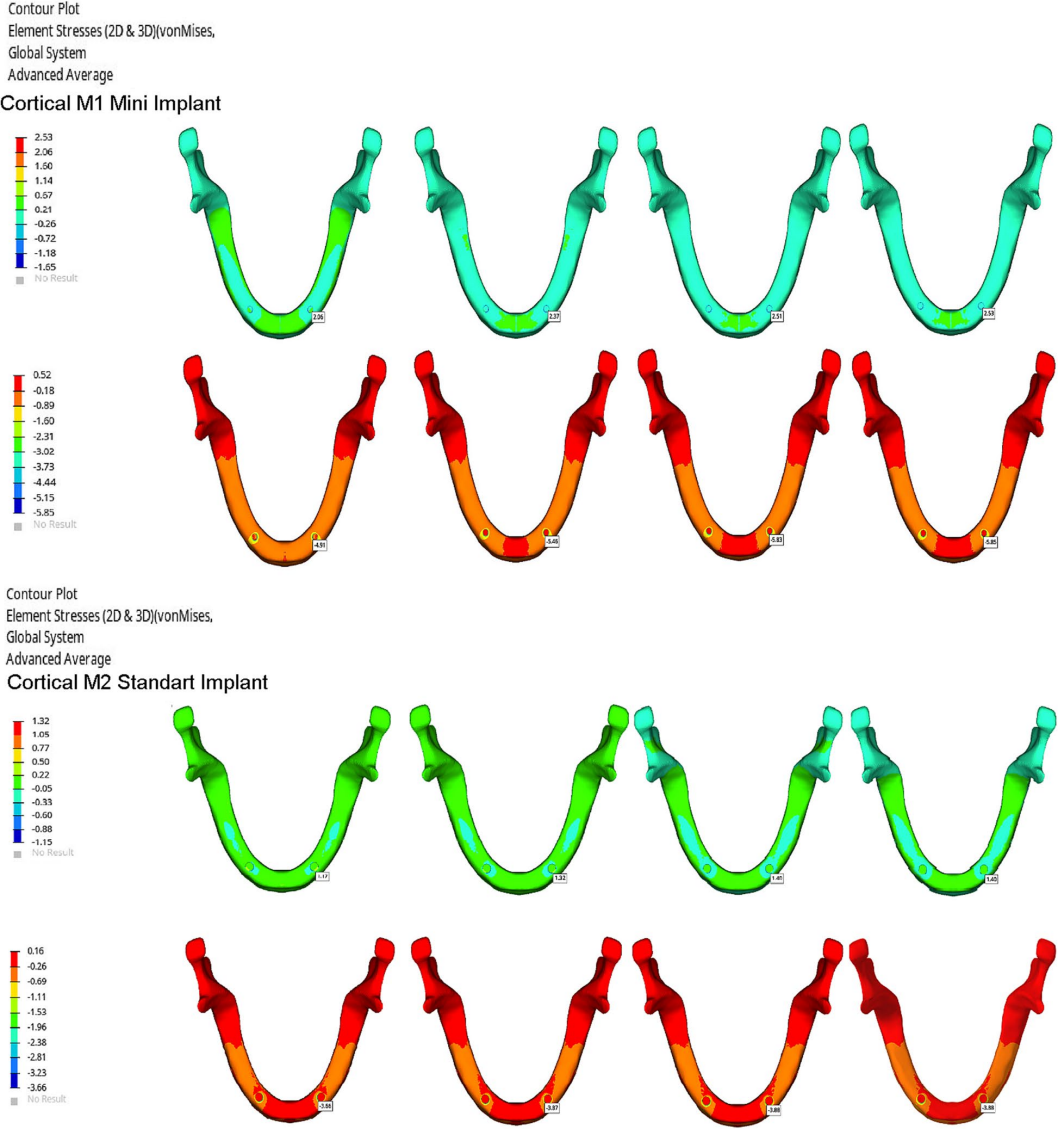
Maximum and minimum principle streses (N/mm2) of cortical bone

**Fig. 6:**
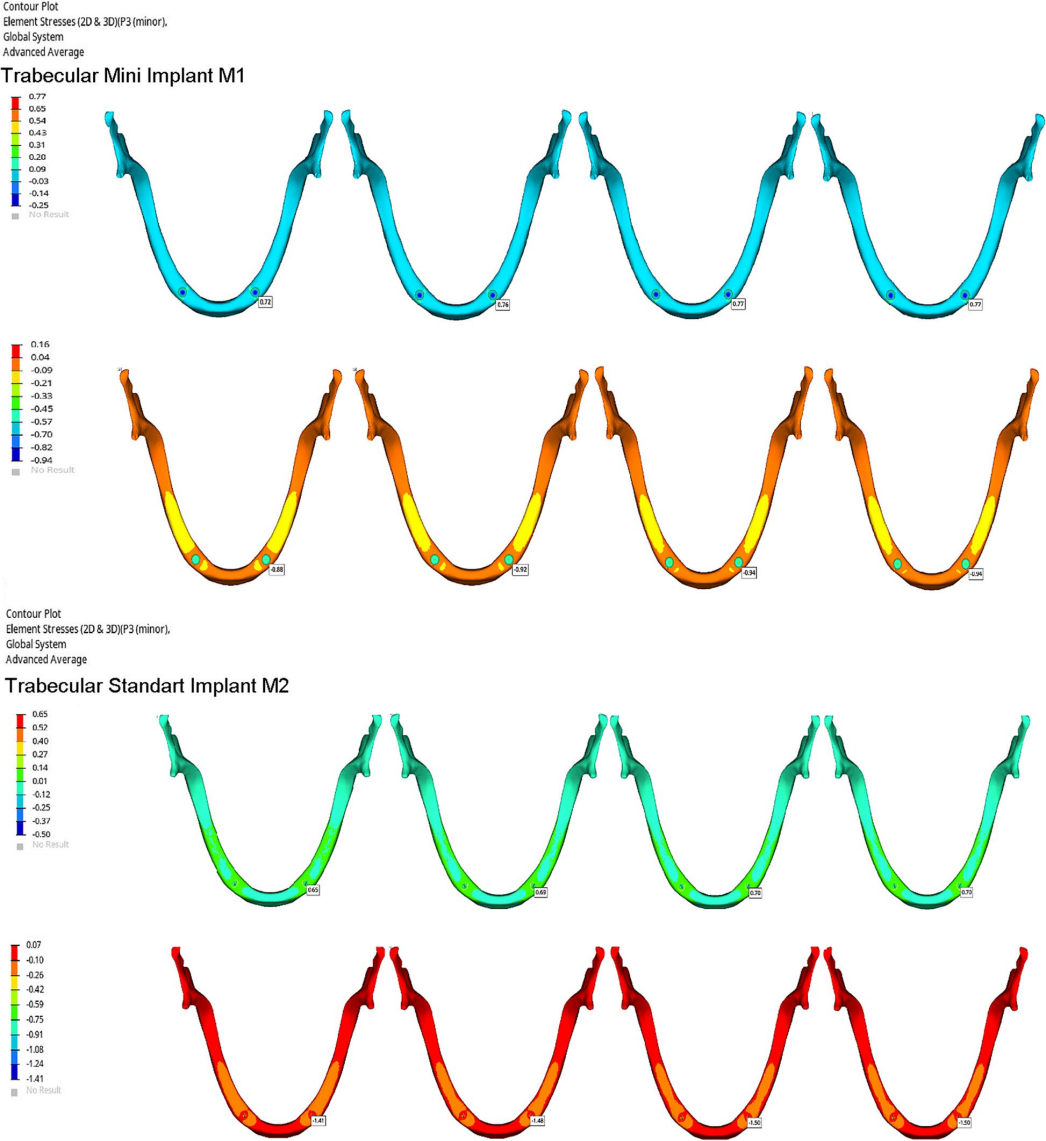
Maximum and minimum principle stresses (N/mm2) of trabecular bone

Stress values on implants, framework materials, and cortical and trabecular bone are shown in Figs. 3, 4, 5 and 6. Tables 3, 4, 5 and 6.

**Table 3:** Von stress (N/mm2) values on the implants

Implants	CoCr	ZANTEX	PEEK	PMMA
M1	10,53	11,12	11,24	11,25
M2	3,86	4,67	5,23	5,28

**Table 4:** Von stress (N/mm2) values on the substructure materials

Substructure materials	CoCr	ZANTEX	PEEK
M1	13,80	4,63	1,85
M2	13,97	4,80	1,90

**Table 5:** Maximum and minimum principle stress (N/mm2) values on cortical bone

Cortical bone		CoCr	ZANTEX	PEEK	PMMA
M1	max principle	2,06	2,37	2,51	2,53
	min principle	4,91	5,46	5,83	5,85
M2	max principle	1,17	1,32	1,40	1,40
	min principle	3,66	3,87	3,88	3,88

**Table 6:** Maximum and minimum principle stress (N/mm2) values on trabecular bone

Trabecular bone		CoCr	ZANTEX	PEEK	PMMA
M1	max principle	0,72	0,76	0,77	0,77
	min principle	0,88	0,92	0,94	0,94
M2	max principle	0,65	0,69	0,70	0,70
	min principle	1,41	1,48	1,50	1,50

Regardless of which framework was supported, higher stress values were observed in all M1 models compared to M2 models.

The highest stress values on the bone and implants were seen in the M1-PMMA model. This was followed by respectively M1-PEEK, M1-ZANTEX, and M1-CoCr. This sequence is the same for the M2 models: M2-PMMA, M2-PEEK, M2-ZANTEX, and M2-CoCr (Figs. 3, 4, 5 and 6) (Tables 3, 4, 5 and 6). The highest stress values for supporting bone were observed in the anterior mandibula, especially implants neck regions. The highest stress value on the implants occured in M1-PMMA (11.25 N/mm2) at the neck of implant. The lowest stress value on the implants was seen in M2-CoCr (3.36 mm/N2)at the neck of implants, as seen the Fig. 3.

Regardless of the model, the highest stress values on the framework materials were observed on CoCr, ZANTEX, PEEK frameworks, respectively. While the stresses on PEEK frameworks were observed at high values especially around the implants, the stresses on Crco substructures spread throughout the structure. The highest stress value was observed CoCr alloy in M2-CoCr (13,97 N/mm2) and the lowest stress value was observed PEEK in M1-PEEK (1,85 N/mm2) (Fig. 4) (Table 4).

## Discussion

The null hypothesis was rejected because it was found that using polymers as prosthetic framework materials resulted in a disadvantageous stress transmission compared to metal frameworks. Regardless of implant size, dentures supported with Zantex transmitted less stress to the bone than dentures supported with PEEK. The highest stress values in supporting bone and implants were observed in PMMA prostheses without framework material. In addition, parallel to our null hypothesis, this study revealed that prosthetic models supported with standard created lower stress values in the bone when compared to models created with mini-implants implants in mandibular overdenture treatments.

Completely edentulous patients often suffer from inadequate retention and stability in their mandibular prostheses. Most of these patients preferred over-implant prosthesis as an economical, aesthetically acceptable, and a feasible treatment method. Today, the use of mini-implant overdentures is becoming a quick and technically easier alternative to traditional implant overdentures [1, 2]. Lemos et al. [20] reported in their systematic review that four mini-implant overdentures may be an alternative treatment option for patients who cannot undergo standard implants, considering the high survival rates, acceptable marginal bone loss, and patient satisfaction rates. Pasini et al. [18] evaluated the stresses on the supporting tissue and implants in single- and standard two-implant-supported overdenture prostheses in a FEA study and argued that there was more movement under chewing forces in mini-implant supported overdenture prostheses, but less stress on them. Souza et al. [21] observed that implant survival rates were respectively 82%, 89%, and 99%, respectively, in overdenture treatments supported by respectively two mini-implants, four mini-implants, and two standard implants, respectively. Enkling et al. [22] suggested that overdenture prostheses supported by four mini-implants are a minimally invasive and economical treatment alternative that improves chewing function and quality of life in patients having mandibular ridges with reduced bone support. Solberg et al. [1] compared overdenture prostheses supported by two standard implants and four to five mini-implants in terms of stress distribution in the mandible. They stated that the resulting stress values ​​were below the critical limits, regardless of the number and type of implants. By contrast, Chang et al. [4] compared the mechanical effects of overdenture prostheses supported by four mandibular mini-implants and two standard implants on bone, and their results indicated that the stress occurring around the implant in overdenture prostheses supported by mini-implants was higher than critical values. Patil et al. [2] biomechanically compared two standard and two mini-implant-supported locator attachment overdenture prostheses in the mandible, and they observed that mini-implant-supported overdenture prostheses placed approximately twice as much stress on the mandibular bone compared to standard implants. In the current study, similar to these publications, significantly higher stress values ​​were observed in the implants and supporting bone, regardless of the framework material of the prosthesis, under chewing forces in overdenture prosthesis models supported by mini-implants. Protesta et al. [23] evaluated overdenture prostheses retained by mini-dental implants (MDIs) as a treatment option for complete edentulism during a 3-year follow-up period, in which MDI overdentures applied to the mandible had better survival rates and health status than those applied to the maxilla. However, they found serious prosthetic complications such as overdenture base fracture, matrix detachment, and instability of the maxillary antagonist prosthesis. Fractures are usually caused by bending fatigue and impact. Continuous exposure to chewing pressure causes fatigue in the acrylic base, which creates microcracks in the polymer. Microcracks enlarge over time and cause failure of the base [17, 24]. The abutment neck of the implant is also the region with the most fractures in implant supported overdenture prostheses. This can be explained by the insufficient thickness of the overdenture base at the fulcrum [25, 26]. Even with a small denture base thickness, metal bases with their long axis placed perpendicular to the static forces strengthen the flexural properties of the prosthesis. Therefore, the denture base supported by metal alloys can prevent base fractures in clinical use [9, 27]. Reinforcement of the mandibular implant-supported overdenture has been suggested as a method to increase fracture resistance and improve the denture's dimensional stability [26]. It has been reported that this approach can reduce stress on implants. In addition, it has been reported that the denture base supported by rigid metal spreads the chewing forces more evenly on the alveolar crest. In addition, metals are used as framework material in overdenture prostheses, and the stresses on the implants can be reduced [26, 28]. The use of CoCr frameworks in mandibular overdenture prostheses has been found to be positive in terms of the distribution of stress on implants and bone [26]. However, metal framework materials are heavy, their adhesion to the acrylic base is not strong, the construction stages are laborious, and there is a possibility of allergy in some cases [27].

Ameral et al. [17] reported that overdenture prostheses supported with CoCr alloy create 62% less stress on the attachment implant and bone compared to unsupported acrylic bases in a FEA study performed on single-implant overdenture prostheses. Gomes et al. [29] compared Ti and stainless steel implant analogs and argued that the higher the elastic modulus of the stainless steel analogs, the lower the stress. Durand et al. [30] compared the restorations they made with inlay materials with different modulus of elasticity with FEA, claiming that materials with high elastic modulus created more stress in the cavity. Materials with a high elastic modulus tend to accumulate stresses on themselves, while materials with a lower elastic modulus tend to transfer stresses to neighboring materials with a higher elastic modulus [29, 30]. In parallel, higher stress values were observed on the CoCr framework material with the highest elastic modulus compared to the PEEK and Zantex framework in this study. In addition, the stress values occurring in the supporting bones and implants in the M1-CoCr and M2-CoCr models are the lowest. The study also observed that the CoCr framework material, with its high elastic modulus, absorbs the stresses and transmits less stress to the implants and bone.

Kelkar et al. [31] used PEEK, Zirconia, and Titanium materials as a framework for prostheses and compared the stress distribution in the supporting tissue. They reported that the Zirconia frameworks provided the best stress distribution. They attributed this result to the high deformation of PEEK material due to its low elastic modulus. Similarly, in a study comparing Ni–Cr and PEEK as framework material, higher values ​​were observed in PEEK-supported prostheses over supporting bone and implants [32]. Diego et al. [33] conducted a biomechanical evaluation of Zirconia, PEEK, carbon fiber, and titanium framework materials in mandibular fixed implant-supported prostheses. They reported that unsupported PMMA- and PEEK-supported prostheses transmit stress on the bone at critical values ​​and provide more successful results than titanium- and Zirconia-supported prostheses. In the study, the highest stress values ​​were observed in the M1M2-PMMA models. The M1-M2 PMMA models gave similar stress results to M1-PEEK and M2-PEEK. This behavior may be related to the fact that the elastic modulus of PEEK material is closer to PMMA compared to other framework materials, and the framework material used in the study is 0.5 mm thin. Since there are no studies in the literature where Zantex is evaluated as a framework material in a removable prosthesis, we could not find the opportunity to discuss this material.

Although many previous studies on framework materials used in implant-supported prostheses support the results of this study [14, 20, 30–33] some studies have argued opposite results [7, 11, 34, 35]. In a photoelastic stress analysis study conducted by Anehosur et al. [7], they compared the use of only heat cured acrylic, Ni–Cr, PEEK, and fiber mesh supported acrylic as a base in two-implant overdenture prostheses and highlighted that the overdenture prosthesis supported with PEEK material gave the best results in terms of stress distribution. In a clinical study by Kortam et al. [34] which compared metal and PEEK material as framework material in maxillary overdenture prostheses, the survival rates of implants supporting PEEK-based overdenture prostheses were found to be higher. Zoidis et al. [35] in a clinical report that compared PEEK and metal alloys mandibular overlay dentures argued that PEEK was more advantageous with lower stress transmission to the teeth due to the elasticity modulus of PEEK being similar to dentin. Frank et al. [11] compared PEEK, ZANTEX, and Ni–Cr as framework material for a fixed implant-supported prosthesis in osteoporotic and normal bone models. Their results showed that PEEK and Zantex provided better results than Ni–Cr. These differences can be explained by the use of different types of prostheses, the use of different bone variants, and the use of different stress measurement methods. Since we could not find a similar study comparing framework materials in implant supported overdentures in the literature, we could not find the opportunity to completely compare our results.

The limitations of finite element studies [17–19, 30, 31, 33, 36] are the assumption that the materials used have isotropic linear elasticity, that the jawbone is homogeneous and implants are 100% osseointegrated into the bone.

Also a clear limitation of the study is that the friction coefficients at the implant and bone interface for the mini-implant and standard implants are set to be similar and zero. Although not seen in a clinical scenario, these are inherent in finite element studies due to limitations in biological simulation. In addition, although there are studies [2, 21] investigating the use of two mini-implants in mandibular implant supported overdentures, there is no clinical background for this indication. Keeping this points in mind, further clinical studies might be considered in this topic.

## Conclusion

Within the limitations of this FEA study, the following conclusions are drawn:

1. Rigid framework materials concentrate the chewing forces and transmit less stress to the supporting tissue and bone in implant supported overdenture prostheses.
2. Mini-implants produce significantly higher stress values in the supporting tissues and implant neck than standard implants.
3. The results of the study suggest that the use of overdenture prostheses formed with rigid framework materials supported by standard implants is more successful.

## Data Availability

The data sets used and/or analysed during the current study are available from the corresponding author on reasonable request.

## Abbreviations

FEA: Finite element analysis
CoCr: Cobalt Chromium alloy
PEEK: Poly-ether ether ketone
PMMA: Poly-methyl metacrilate
